# The *EDN1* Missense Variant rs5370*G > T* Regulates Adaptation and Maladaptation under Hypobaric Hypoxia

**DOI:** 10.3390/ijerph191811174

**Published:** 2022-09-06

**Authors:** Tsering Palmo, Bilal Ahmed Abbasi, Neha Chanana, Kavita Sharma, Mohammed Faruq, Tashi Thinlas, Malik Z. Abdin, Qadar Pasha

**Affiliations:** 1 Genomics and Molecular Medicine, CSIR-Institute of Genomics and Integrative Biology, Delhi 110007, India; 2 Department of Biotechnology, Jamia Hamdard, New Delhi 110062, India; 3 Sonam Norboo Memorial Hospital, Leh 194101, Ladakh, India; 4 Institute of Hypoxia Research, New Delhi 110067, India

**Keywords:** high altitude, hypobaric hypoxia, adaptation, endurance, maladaptation, high-altitude pulmonary oedema, endothelin 1, vascular homeostasis

## Abstract

Endothelin 1 (*EDN1*) encodes a potent endogenous vasoconstrictor, ET1, to maintain vascular homeostasis and redistribution of tissue blood flow during exercise. One of the *EDN1* missense polymorphisms, rs5370 *G/T*, has strongly been associated with cardiopulmonary diseases. This study investigated the impact of rs5370 polymorphism in high-altitude pulmonary oedema (HAPE) disorder or maladaptation and adaptation physiology in a well-characterized case–control study of high-altitude and low-altitude populations comprising 310 samples each of HAPE-patients, HAPE-free controls and native highlanders. The rs5370 polymorphism was genotyped, and the gene expression and plasma level of EDN1 were evaluated. The functional relevance of each allele was investigated in the human embryonic kidney 293 cell line after exposure to hypoxia and computationally. The T allele was significantly more prevalent in HAPE-p compared to HAPE-f and HLs. The *EDN1* gene expression and ET1 bio-level were significantly elevated in HAPE-p compared to controls. Compared to the G allele, the T allele was significantly associated with elevated levels of ET-1 in all three study groups and cells exposed to hypoxia. The *in silico* studies further confirmed the stabilizing effect of the T allele on the structural integrity and function of ET1 protein. The ET1 rs5370 T allele is associated with an increased concentration of ET-1 in vivo and in vitro, establishing it as a potent marker in the adaptation/maladaptation physiology under the high-altitude environment. This could also be pertinent in endurance exercises at high altitudes.

## 1. Introduction

High altitude (HA, above 2500 m) is characterized by hypobaric hypoxia, a stressful environmental condition with a low atmospheric barometric pressure which leads to reduced inspired oxygen at the cellular level. HA is one of the natural habitats colonised by humans several thousand years ago [1]. The clinical, transcriptomic, proteomic and in silico studies in natives of Tibet, Ladakh, the Andean Altiplano, and the Ethiopian highlands have revealed independent and unique adaptive traits in each population [2,3,4,5]. If they fail to acclimatise, sojourners who travel to HA suffer from fatal mountain disorders such as high-altitude pulmonary oedema (HAPE). The pathophysiology of HAPE involves remodelling endothelial and vascular smooth muscle cells (VSMCs), which leads to uneven pulmonary vasoconstriction, resulting in oedema in the alveoli regions of the lungs [6,7,8].

The endothelium regulates vascular tone by releasing numerous vasoconstrictors and vaso-relaxants. Among the several vascular tone markers contributing to homeostasis, endothelin-1 is one potent marker [9]. Vascular endothelium and VSMCs of lungs, the two main sites of ET-1 production, are strongly associated with pulmonary vasculature. The endothelin-1gene (*EDN1*), located on chromosome 6, encodes the endothelin (ET) peptide. Out of its three isoforms, ET-1, ET-2, and ET-3, ET-1 is the most prevalent isoform in the cardiopulmonary system, formed after the sequential proteolytic cleavage of pre-pro-ET-1 (ppET-1) [10]. The binding of ET-1 to the ET_A_ receptor on smooth muscles induces vasoconstriction. In contrast, the binding of ET-1 to the ET_B_ receptor on endothelial cells stimulates nitric oxide production to elicit vasorelaxation [11]. Hypoxia and shear stress induces the binding of hypoxia-inducible factor-1α (HIF1α) to the hypoxia-responsive element of *EDN1* located in the promoter region to upregulate the expression of *EDN1* [12,13]. Increased ET1 levels also play an essential role in exercise-induced tissue blood flow and perfusion redistribution in athletes [14,15,16]. HIF1α is a transcriptional factor responsible for regulating adaptive responses to hypoxia by activating the transcription of many genes, including *EDN1* [17,18]. Several *EDN1* polymorphisms strongly correlate with *EDN1* expression and plasma ET-1 levels in cardiovascular and pulmonary hypertension patients [19,20,21]. Of these, the missense variant rs5370 *G/T* present on the ppET-1 and encoding Lys198Asn (K198N) has received greater attention owing to its strong association with pulmonary arterial hypertension, cardiovascular diseases, HAPE, and cancers [19,20,21,22,23]. Albeit this polymorphism’s role in cardiovascular, hypertension and various cancers has been widely explored, its cellular role in HAPE or HA adaptation is yet to be explored.

Therefore, in the present study, we have investigated the genotype and allele distribution of the polymorphism rs5370 *G/T* in a large sample size of HAPE patients (HAPE-p), HAPE-free controls (HAPE-f) and native highlanders (HLs). The expression of *EDN1* and plasma level of ET-1 were estimated in the human samples. To validate the cellular effect of the variant, ET-1 expression was evaluated using a human embryonic kidney cell line 293 (HEK293) under normoxic and chemically induced hypoxic conditions. In addition, we have performed an extensive in silico analysis to understand the impact of the allelic change on the protein’s structure and function using a series of bioinformatics tools.

## 2. Materials and Methods

### 2.1. Study Subjects

In the current study, the subjects were categorized into three well-defined groups: (1) HAPE-p, (2) HAPE-f of Indo-Aryan ethnicity, and (3) HLs, the native of Ladakh of Tibeto-Burman ethnicity. Further to elaborate, (i) HAPE-p were sojourners who suffered HAPE upon exposure to HA (3500 m); (ii) HAPE-f were the healthy sojourners who visited HA under similar conditions but did not suffer from the HAPE; and (iii) HLs were permanent residents of altitude at and above 3500 m for many generations. HAPE-p, HAPE-f and HLs, numbering 310 subjects each, were recruited through Sonam Norboo Memorial (SNM) hospital, Leh (3500 m), Ladakh, India.

### 2.2. Clinical Characteristics

Age, height, weight, body mass index (BMI), and clinical characteristics, such as systolic blood pressure (SBP), diastolic blood pressure (DBP), arterial oxygen saturation (SaO_2_), and pulse rate (PR) of each subject, were recorded. Informed and written consent was obtained from each subject prior to their participation in the study. The human ethical committees of Council of Scientific and Industrial Research Institute of Genomics and Integrative Biology, Delhi, and SNM Hospital, Leh, Ladakh, had approved the investigation.

The diagnosis of HAPE was based on SaO_2_ levels, the presence of pulmonary rales, cyanosis, and chest X-ray. The clinical symptoms included cough, dyspnoea at rest, breathlessness, absence of infection, and reduced exercise performance. SaO_2_ levels were measured using a Finger-Pulse Oximeter 503 (Criticare Systems, Surat, India). The blood pressure was measured using automatic blood pressure monitor HEM-7156 (Omron, Kyoto, Japan). The mean arterial pressure (MAP) was calculated using 1/3 × SBP + 2/3 × DBP formula. Approximately 10 mL venous blood sample was drawn in acid-citrate-dextrose anticoagulant tubes. Plasma and peripheral blood leucocytes were separated. Plasma and RNA were stored at −80 °C and DNA at −20 °C.

### 2.3. Selection and Genotyping of Polymorphism

Through a literature survey, several polymorphisms of *EDN1* were evaluated for their functional relevance, position in the gene, i.e., exonic, and use in previous studies; among these, the missense SNP rs5370 *G/T* emerged as the most relevant. The polymorphism was genotyped in 310 subjects of each study group using the SNaPshot ddNTP Primer Extension PCR kit (Applied Biosystems, Waltham, MA, USA). For genotyping, primers were designed using Primer3 software, Primer3 Input Available online: https://primer3.ut.ee/ (accessed on 3 March 2022). The primer sequence and optimal conditions for amplification are presented in Supplementary Table S1.

### 2.4. Quantitative RT-PCR

Gene expression was performed on 40 samples each of HAPE-p, HAPE-f, and HLs. Total RNA was extracted from an aliquot of 2 mL whole blood by TRI reagent RT blood (Molecular Research Centre, Cincinnati, OH, USA). RNA quantity and quality were determined on a NanoDrop ND-1000 spectrophotometer, and integrity was checked by running on 1.5% agarose gel. Total RNA, 5.0 μg, was used to generate cDNA by RevertAid First Strand cDNA Synthesis Kit (Thermo Scientific, Waltham, MA, USA) for real-time polymerase chain reaction (qRT-PCR) of several genes. The primers for qRT-PCR were designed using the NCBI primer BLAST browser. The primers and optimal conditions for amplification are presented in Supplementary Table S2. qRT-PCR was performed in triplicate for each gene and each sample on LightCycler 480 RT-PCR System (Roche Molecular Diagnostics, Pleasanton, CA, USA) using MESA BLUE qPCR Master Mix Plus for SYBR^®^ Assay No ROX (Eurogentec, Seraing, Belgium). Relative transcript quantity was calculated using the ΔΔCt method with 18SrRNA as an endogenous reference gene.

### 2.5. Site-Directed Mutagenesis of EDN1

The *EDN1 Asn198* variant was created using Quick Change II XL Site-Directed Mutagenesis Kit (Agilent, Santa Clara, CA, USA). Briefly, the wild-type (WT) *EDN1* Lys198 plasmid (NM_001955, Origene Technology Inc., Rockville, MD, USA) was amplified using a pair of synthetic oligonucleotide primers containing the desired variant. The mutated primers used were as follows (the variant sequence is underlined): forward primer, 5’-cccaagctgaaaggcaatccctccagagagc-3’; G594Treverse primer, 5’-gctctctggagggattgcctttcagcttggg-3’. The PCR amplicon representing variant plasmid with staggered nicks was treated with *Dpn1* and transformed into XL1-blue super competent cells. Generation of the variant ET1 plasmid encoding Asn198 was confirmed by sequencing with the universal primer M13R-pUC-26, 5′-CAGGAAACAGCTATGAC-3′.

### 2.6. Cell Culture, Transient Transfections and Hypoxia Treatment

Human embryonic kidney 293 (HEK293) cells (National Centre for Cell Sciences, Pune, India) were cultured in DMEM (Hi media, West Chester, PA, USA) supplemented with 10% (*v*/*v*) foetal bovine serum (Thermo Fisher Scientific, Waltham, MA, USA) and 5% CO_2_ at 37 °C in a humidified incubator. A total of 1× antibiotic-antimycotic (Thermo Fisher Scientific, Waltham, MA, USA) was used to prevent contamination. HEK293 cells were either transfected with one µg of pcDNA3.1 empty vector, one µg of WT *EDN1* Lys198 or one µg of Var *EDN1* Asn198using Lipofectamine 3000 (Thermo Fisher Scientific, Waltham, USA). Each of these transfected cells was either kept under normoxic conditions for 30 h or was given hypoxia treatment, 6 h post-transfection, using 100 µM cobalt chloride for 24 h. Thirty hours post-transfection, media were collected for ET1 estimation.

### 2.7. Quantification of Circulating Plasma and Cellular ET-1 Levels

The plasma ET1 levels of 60 subjects in each of the three study groups and cellular ET1 levels were measured by enzyme immunoassay-basedET1 kit (USCN Life Science, Wuhan, China) on a high-throughput SpectraMax plus384 Spectrophotometer (Molecular Devices, San Jose, CA, USA), according to manufacturer’s instructions.

### 2.8. Statistical Analyses

Unpaired Student’s *t*-test (two-tailed) was performed to compare the differences in clinical parameters and bio-levels between the two groups and cellular ET1 levels. Hardy–Weinberg equilibrium was checked using the χ2 goodness-of-fit test. Genotype and allele distributions, odds ratio (OR), and 95% confidence interval (CI) were calculated by multinomial logistic regression (MLR) using SPSS-15.0. Single locus analysis was performed to determine the association of SNP with either HAPE susceptibility or adaptation using co-dominant, dominant, recessive, and allelic models. In the co-dominant model, heterozygous *GT* and recessive homozygous *TT* are compared to the wild homozygous *GG*. The dominant model compared *GG* versus *GT* + *TT*, and a recessive model compared *GG* + *GT* versus *TT*. The allelic model estimated the association at allelic level i.e., G versus T. The covariates were age and BMI. One-way ANOVA analysed the quantitative RT-PCR data. The allele association with ET-1 level was performed using an unpaired Student’s *t*-test. A *p* value < 0.05 was considered statistically significant.

### 2.9. In Silico Analyses

#### 2.9.1. Data Collection

The sequence information of ppET-1 protein was obtained from the Uniprot database (ID: P05305). The 3D structural model was retrieved using Alphafold v2.1.14 as no complete 3D structure was available [24]. Further, the functional annotation of rs5370 polymorphism was deciphered using sequence-based analysis, aggregation propensity, conservation, frustration analysis, and simulation studies.

#### 2.9.2. Sequence-Based Analysis

SIFT [25], PROVEAN [26], and PolyPhen-2 [27], the sequence-based tools, were used to identify the impact of missense variant of SNP rs5370 on the structure and function of ppET-1.

#### 2.9.3. Aggregation Propensity, Conservation and Frustration Analysis

SODA, an online tool, was employed to determine the change in protein solubility due to the missense variant [28]. The degree of amino acid residue conservation impacts a protein’s structure and function therefore, ConSurf was employed to identify amino acid residues’ conservation degree [29]. Further, the Frustratometer web tool was used to assess the residual frustration in the ppET-1 protein [30]. In the output, Z value < 0.78 indicates the variant to be highly frustrated, while >0.78 is minimally frustrated towards the protein.

#### 2.9.4. Molecular Dynamics Simulations

The 3D structure of ppET-1, retrieved from Alphafoldv2.1.14, was further refined and prepared using the protein preparation wizard with the Schrodinger suite [31]. Additionally, the mutation in the protein structure was induced by Pymol mutagenesis wizard. The energy minimization was performed for the ppET-1 structures with the wild-type allele G and variant type allele T of SNP rs5370 using the Optimized Potentials for Liquid Simulations (OPLS)-2005 force-field along with 0.30Å as a cut-off value. Both structures were used for separate, all-atom MD simulations with 100 ns run in Groningen Machine for Chemical Simulations (GROMACS) 2020.2 using the OPLS force field [32]. The protein system initiated in a cubic box was solvated using the TIP-3P water representation [33]. The initial structure was subjected to energy minimization using the steepest descent method, simulated at 300 K (Kelvin) using a modified Berendsen thermostat and further subjected to a Parrinello–Reman barostat for pressure coupling at 1 bar [34]. Additionally, the electrostatic interactions were determined by the particle mesh Ewald method [35]. The time step was two femtoseconds, and a leap-frog integrator was applied for the time evolution of trajectories. Further, the trajectory files were evaluated using GROMACS embedded modules and Visual MD software.

## 3. Results

### 3.1. Clinical Assessment Showed Significant Variations in Patients

**Figure 1:**
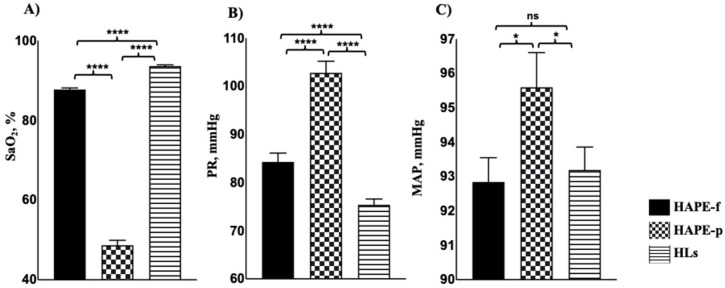
Clinical characteristics of the three study groups. Among the clinical parameters, (**A**) SaO_2_ levels were lower in the HAPE-p (*n* = 310) compared to the HAPE-f (*n* = 310) and HLs (*n* = 310) (*p* < 0.001); (**B**) PR was elevated in HAPE-p compared to HAPE-f and HLs (*p* < 0.001), and (**C**) MAP was also elevated in HAPE-p compared to HAPE-f and HLs (*p* < 0.05). Data are presented as mean ± SE and are compared by a two-tailed *t*-test. * *p* < 0.05, **** *p* < 0.0001 was considered statistically significant. ns, non-significant; SE, Standard error.

The SaO_2_ level in the HAPE-p was 48.6 ± 13.5%, which was significantly lower compared to the levels of 87.7 ± 4.4% and 93.6 ± 2.8% in the two healthy groups, i.e., HAPE-f and HLs (*p* = 4.71 × 10^−77^ and 3.92 × 10^−65^, respectively; Figure 1A). The PR in the HAPE-p was 102.8 ± 21.9 rate/min, which was significantly higher compared to 84.2 ± 20.4 rate/min and 75.4 ± 10.6 rate/min in the two healthy groups, HAPE-f and HLs (*p* = 6.38 × 10^−9^ and 5.76 × 10^−18^, respectively, Figure 1B). The mean arterial pressure (MAP) in HAPE-p was 95.6 ± 10.7 mmHg, which was significantly higher compared to 92.8 ± 7.7 mmHg and 93.2 ± 6.91 mmHg in the two healthy groups, HAPE-f and HLs, respectively (*p* = 0.02, each; Figure 1C). All levels are reported as the mean ± SD (standard deviation) (Supplementary Table S3).

### 3.2. Genotype and Allele Distribution Differed in Patients and Healthy Subjects

The control groups were in Hardy–Weinberg equilibrium for the studied SNP. The heterozygote genotype rs5370 *G/T* was most prevalent in HAPE-p compared to HAPE-f and HLs (*p* = 1.75 × 10^−5^ and 1.78 × 10^−9^, respectively). The MLR analysis revealed a significantly higher risk of HAPE with rs5370T when compared with HAPE-f and HLs, (*p* = 0.002 and 3.09 × 10^−6^, respectively); hence, rs5370T was recognized as a risk allele, whereas the rs5370*G* was overrepresented in healthy controls, and was recognized as a protective allele towards HAPE. Furthermore, the HLs compared to HAPE-f were overrepresented with the genotype combination *G/G* and *G/T* (*p* = 0.04), inferring the role in adaptation (Table 1).

### 3.3. Elevated ET-1 Expression and Protein Levels Associated with HAPE

**Figure 2:**
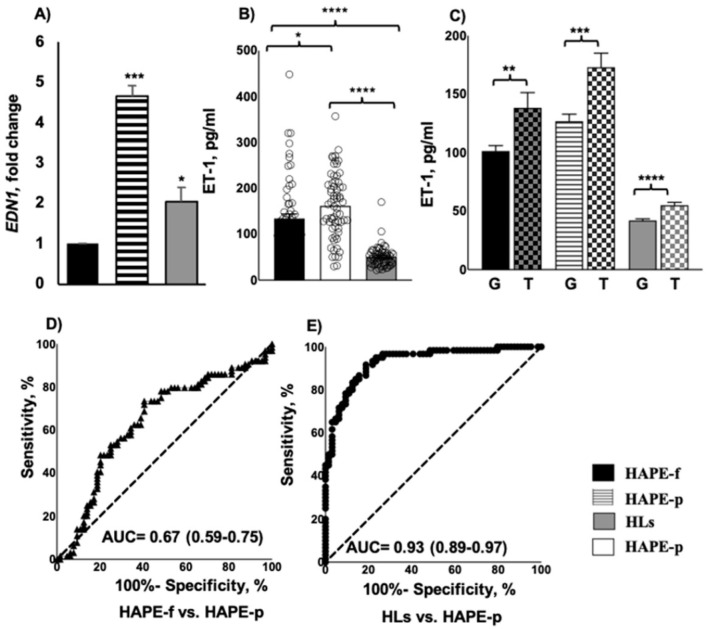
*EDN1* gene expression and ET1 plasma levels in the three study groups. (**A**) The relative expression of *EDN1* was evaluated by RT PCR. The *EDN1* expression was upregulated by 4.7-fold and 1.8-fold in the HAPE-p (*n* = 40) and HLs (*n* = 40) compared to HAPE-f (*n* = 40) (*p* < 0.001 and *p* = 0.03, respectively). (**B**) Plasma level of ET1, pg/ml was evaluated by immunoassay. ET-1 level was significantly higher in HAPE-p (*n* = 60) compared to HAPE-f (*n* = 60) and HLs (*n* = 60) (*p* = 0.03 and <0.001, respectively); (**C**) the risk allele T associated with the higher concentration of ET-1 in the three study groups compared to protective G allele (*p* < 0.05). (**D**,**E**) ROC curve exhibiting the significant AUC of 0.67 (0.59–0.75) and 0.93 (0.89–0.97) demonstrates the effect of ET-1 levels in HAPE-p (*p* < 0.001). Data are presented as mean ± SE and are compared by a two-tailed *t*-test. * *p* < 0.05, ** *p* < 0.01, *** *p* < 0.001, **** *p* < 0.0001 was considered statistically significant. ROC, receiver operating characteristic curve; AUC, area under the curve; SE, standard error.

*EDN1* expression was upregulated by 4.7-, and 1.8-fold in the HAPE-p and HLs compared with the HAPE-f (*p* = 6.05 × 10^−7^ and 0.03, respectively; Figure 2A). The ET-1 level of 161.98 ± 70.50 pg/mL was significantly higher in HAPE-p as compared to the levels of 134.46 ± 79.20 pg/mL and 51.11 ± 22.70 pg/mL in the two healthy groups, HAPE-f and HLs (*p* = 0.04 and 3.76 × 10^−12^, respectively, Figure 2B). The risk T allele is associated with the higher concentration of ET-1 in HAPE-f, HAPE-p and HLs (*p* = 0.009, 0.001 and 7.96 × 10^−5^, respectively, Figure 2C).

The receiver operating characteristic curve (ROC) exhibited significant area under the curve (AUC) with values of 0.67 (0.59–0.75) for HAPE-f vs. HAPE-p (Figure 2D) and 0.93 (0.889–0.97) in HLs vs. HAPE-p (Figure 2E) to demonstrate the significantly increased level of ET-1 in HAPE-p (*p <* 0.001).

### 3.4. Allele rs5370T Associated with Enhanced Cellular ET1 Levels under Normoxia and Hypoxia

**Figure 3:**
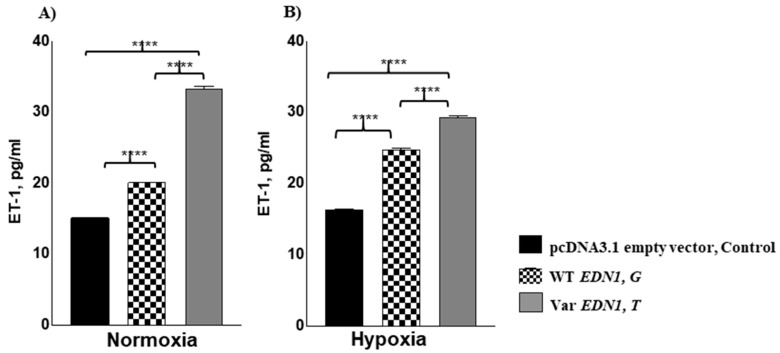
Both hypoxia and *EDN1* rs5370*T*/Asn198 variant enhanced cellular ET1 levels. HEK293 cells were either transfected with one µg of pcDNA3.1 empty vector, control, or one µg of WT *EDN1* G allele expressing plasmid or with one µg of Var *EDN1* T allele expressing plasmid. Each of the transfected cells was either kept under (**A**) normoxic conditions for 30 h or (**B**) normoxia for 6 h, followed by hypoxic conditions (100 Μm CoCl_2_) for 24 h. Secreted ET-1 levels were highest in the presence of the risk T allele under normoxia and hypoxia. Data are presented as mean ± SE and are compared by a two-tailed *t*-test. **** *p* < 0.0001. WT, wild-type allele; Var, variant-type allele; SE, standard error.

HEK293 cells were transfected with the construct having SNP5370 risk T allele which enhanced the secreted ET-1 level both under normoxia as well as hypoxia (100 µM CoCl_2_/24 h; Figure 3). Under normoxic conditions, HEK293 cells transfected with the risk T allele, enhanced secreted ET1 levels by 18 pg/mL while the protective G allele enhanced the ET1 levels by four pg/mL compared to the empty vector that served as control (*p* = 6.22 × 10^−5^ and 2.11 × 10^−6^, respectively; Figure 3A). Furthermore, under chemically induced hypoxia, HEK293 cells transfected with the risk T allele enhanced extracellular ET1 levels by 13 pg/mL while the protective G allele improved ET1 levels by eight pg/mL compared to control (*p* = 1.51 × 10^−6^ and 1.12 × 10^−5^, respectively; Figure 3B). Thus, the risk T allele compared to G allele, increased the cellular ET1 levels under both normoxia and hypoxia (*p* < 0.001).

### 3.5. Sequence-Based Analysis Favours a Deleterious Effect of the Variant Allele

The sequence-based tools computed the deleterious effect of the variant. Sorting intolerant from tolerant (SIFT) and polymorphism phenotyping v2 (Polyphen-2) indicated that the Lys198Asn or K198N (rs5370*G/T*) amino acid change has a deleterious effect with a score of 0.025 and 0.454, respectively. The protein variation effect analyser (PROVEAN) score was −0.890, which indicated a neutral impact on the function of the ppET-1 protein.

### 3.6. Aggregation Propensity, Conservation and Frustration Analyses

Solubility based on disorder and aggregation (SODA) predicted the increased solubility of ppET-1 in the presence of the variant 198Nwith the SODA score of 2.35, aggregation score of 1.61, and disorder score of 0.005. The conservation analysis revealed the amino acid stretches in ppET-1, ranges 51–93,111–124, and 184–188 as highly conserved, and sequence stretches 30–48, 98–108, 125–131, and 138–148 as less conserved. The variant was located in the variable range conferring the variant as less conserved (Supplementary Figure S1). The local frustration indices revealed that the C-terminal residues (175–213) housing the variant sequence increased the frustration compared to the wild-type sequence (Supplementary Figure S2). These observations suggest that the Lys198Asn (rs5370*G/T*) amino acid change is more active, affecting the protein levels.

### 3.7. Molecular Dynamics Simulations Confirmed the Structural Flexibility of 198N Mutation

**Figure 4:**
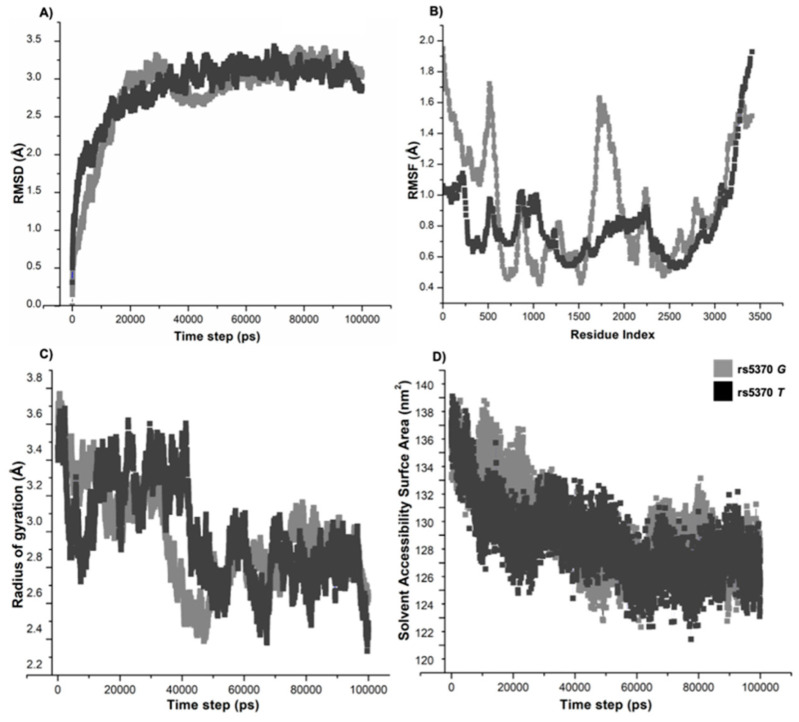
Structural dynamics of ppET-1 for rs5370 alleles. (**A**) RMSD, the molecular dynamics simulation for the wild-type system shows a gradual increase in the RMSD value with more fluctuations compared to variant allele. (**B**) RMSF; the wild-type residue’s fluctuation level was higher than the variant residue’s fluctuation. (**C**) Rg values and variant allele depicted structural stabilizing effects with higher gyration values when compared to the wild type. (**D**) SASA values, the wild type represented higher SASA values as compared to the variant allele. The ordinate is RMSD (Å), and the abscissa is time (ps). Light grey and dark grey indicate wild and variant residues, respectively. Å, Angstrom; nm^2^, nanometre square; ps, picosecond; Rg, radius of gyration.

Molecular dynamics (MD) simulations investigated the impact of the K198N mutation on the conformational stability of ppET1 through 100 ns simulations considering both wild- and variant-type structures. The simulation stabilities for both the protein structures characterized by root-mean-square deviation (RMSD) analysis revealed that both the protein systems attained convergence around 60-ns of simulation. The average RMSD, root-mean-square fluctuation (RMSF), radius of gyration (Rg), solvent accessibility surface area (SASA) and intramolecular hydrogen bond values for both the wild and variant structural models were represented in Supplementary Table S4. Interestingly, the variant198N showed fewer structure deviations over a period of time (Figure 4A). The RMSF plot demonstrated more fluctuations in the wild-type residue compared to the variant 198N (Figure 4B). The average RMSF pattern complimented the RMSD distribution. The average *Rg* values of the wild-type and variant-type models were 2.97 nm and 3.20 nm, respectively. The higher *Rg* of the variant198N confirmed its substantial role in altering the protein conformation by increasing the structural flexibilities, thus affecting the protein stability (Figure 4C). Further, the average SASA values of wild- and variant-type models were found as 130.6 nm^2^ and 129.28 nm^2^, respectively (Figure 4D). The variation198N does not show any significant change in the SASA value compared to the wild-type. Lastly, to understand the stability and flexibility, intramolecular hydrogen bonds formed during the simulation were studied; the wild-type model showed 110 hydrogen bonds, whereas the 198N variant depicted an average of 114 hydrogen bonds during the simulation. Additional findings are presented in Supplementary Table S4.

## 4. Discussion

The present study is the first to comprehensively investigate the functional aspect of rs5370*G/T* polymorphism of *EDN1* using a combined in vivo, in vitro, and in silico approach. The study’s outcome helped to unravel the role of wild and variant alleles in HAPE susceptibility and adaptive physiology in the hypobaric hypoxic environment of high altitude. Our clinical assessment demonstrated a significantly reduced SaO_2_ level and elevated MAP and PR in HAPE-p compared to the two healthy control groups, HAPE-f and HLs. The upregulation of *EDN1* and increased concentration of ET-1 in HAPE-p suggest a role in excessive pulmonary vasoconstriction, which further impairs the vasomotor responses. Conversely, HLs and healthy lowlanders exhibited downregulated expression of *EDN1* and reduced levels of ET-1. ROC analysis performed to determine the influence and association of HA environment on ET-1 levels further supported our observations of increased ET1 levels in HAPE-p compared to control groups.

It has been reported that ET-1 levels are significantly increased in HAPE patients [36,37,38]. In our study, the HAPE-associated increased ET-1 levels correlated with elevated gene expression. Similarly, Ali et al. reported a positive correlation between the circulatory level of ET-1 with the levels of asymmetric dimethylarginine (ADMA) and superoxide dismutase (SOD) in the HAPE-p [39]. The increased circulatory level of ADMA inhibits the synthesis of vasorelaxant nitric oxide synthase and enhances vasopressor angiotensin-1 converting enzyme [8,40]. These variations expectedly contribute to suppressed levels of nitric oxide and enhanced levels of angiotensin II, and thus vascular constriction, which is a hallmark of HAPE and pulmonary hypertension. Reduced ability to ET-1 clearance from hypertensive and diabetic patients was independently associated with higher systolic blood pressure and oxidative stress [41]. The inhibitory effects of ET-1 are not only limited to the dilations induced by NO but also to β-adrenergic receptor-dependent relaxation through activation of the protein kinase C pathway and the ADMA system [42]. Moreover, ET-1 at elevated concentrations is also pro-inflammatory and is pertinent in pulmonary arterial hypertension, HAPE and probably other vascular diseases [43,44,45]. Thus, excessive ET-1 production contributes to the high vascular tone and abnormal proliferation of muscle cells leading to pulmonary vasoconstriction in HAPE-p.

The genotype distribution of the rs5370*G/T* polymorphism and the frequency of the respective alleles revealed a distinct distribution. MLR analysis revealed an overrepresentation of the risk-allele rs5370Tin HAPE-p and the protective G allele in HLs, suggesting the risk and protective roles of the alleles in HAPE pathophysiology. Interestingly, the risk allele T was associated with the increased level of ET-1 in the three study groups. There is ample evidence in the literature of an association between the T allele with various endothelial-associated diseases [46,47]. In contrast, G allele enrichment was associated with adaptation and acclimatisation to the HA environment [36,48]. Significantly higher frequency of the *EDN1 G/G* genotype and G allele in the ethnically different population of HAPE-f and HLs confirmed the genetic role of ET1 in acclimatisation and adaptation at HA. On the other hand, the association of the T allele with enhanced ET-1 levels predisposes individuals carrying T allele to HAPE and other cardiopulmonary diseases. Thus, a distinct role of rs5370 *G/T* allele in protection and risk to HA adaptation or maladaptation, respectively, has been identified.

Hypoxic regulation of *EDN1* is well-known [17,49]. To understand the allelic impact of the widely studied *EDN1* rs5370*G/T* polymorphism in regulating ET-1 under both hypobaric and normoxic conditions, we investigated the allele-specific functional relevance using site-directed mutagenesis, and the outcome was highly encouraging. The endogenous ET-1 levels were significantly upregulated in the chemically induced hypoxic HEK 293 cells transfected with the T allele expressing plasmid. Thus, carriers of the rs5370T allele are associated with increased ET-1 levels under hypoxic cellular conditions and high-altitude associated hypobaric hypoxia, which eventually augment the vascular constriction, and elevate oxidative stress and systolic blood pressure.

Of relevance and interest, in silico studies also confirmed and complemented in vitro and in vivo findings. The pathogenicity of rs5370 was determined using sequence-based, aggregation, conservation and frustration analysis. The atomistic levels of K198N variation compromising the structural integrity were analysed in detail using a 100-ns MD simulation. RMSD, RMSF, Rg, SASA and the hydrogen bond analysis overall indicated higher structural flexibility, reduced fluctuation amongst the protein residues and increased protein compactness, modifying the protein’s dynamic behaviour. The higher structural flexibility of variant allele, resulting in198N amino acid, contributes to enhanced protein stability, aiding in the upregulation of ET1and leading to an increased downstream translational process of ET-1. Thus, the difference in individual alleles affects overall protein structural stability, ultimately affecting protein-protein interactions and the downstream proteolytic cleavage process of ppET-1. However, little is known about the underlying mechanisms of missense variant rs5370 regulating *EDN1* transcription in humans. It is possible that the increased *EDN1* expression may be regulated by the gain of function mutation of the T allele as the localization of this SNP is crucial, which further increases the translation of ET1 protein by virtue of its significant quantitative trait loci. Due to this change, epigenetic factors’ role is inevitable [50,51]. These findings suggest that patients carrying the risk allele T, associated with higher ET-1 levels, are more prone to cardiopulmonary disorders, while the G allele carriers incline towards adaptation.

An interesting scenario is apparent if we attempt to correlate the present findings with physical performances at HA. Normal arterial oxygen levels, as present in the native highlanders, are associated with HA-adaptation and routine physical activities [52]. The athletic performance of the natives has been remarkable compared to the lowland athletes under HA-hypoxia [52,53]. Moreover, pre-acclimatization by hypoxia conditioning prevents HA-associated disorders and maintains the endurance performance of healthy individuals and/or athletes or marathon runners at high-altitude [53,54]. ET-1, on the other hand, is involved in the acute cardiovascular response to hypovolaemia induced by prolonged dynamic exercise [55]. Sympathetic vasoconstriction regulates the vascular tone at rest, but substantial attenuation of the same has been reported in the exercising skeletal muscle vasculature [16]. In such a situation, nonadrenergic vasoconstrictor pathways may contribute significantly to the balance of blood flow in skeletal muscle and thus MAP during exercise. Endothelin-1 is known to elicit a sustained vasoconstrictor response by binding to ET_A_ and ET_B_ receptors on the vascular smooth muscle [13,16]. Thus endothelin-1 seems to be a potential marker in sports medicine.

## 5. Conclusions

This study investigated the allelic effect of rs5370 polymorphism in the HAPE pathophysiology and adaptation physiology at HA. Our results demonstrated a significant association between the rs5370 T allele and levels of ET-1. Functional validation in HEK293 cells also confirmed that the T allele increased ET-1 expression under the influence of hypoxia. The enrichment of the rs5370 G allele in healthy sojourners and the native HA population strongly suggested its role in protection and adaptation at HA. Furthermore, advanced integrated bioinformatics approaches indicated the functional significance of risk allele rs5370 T and its effect on protein structure integrity and function. Overall, the study of the rs5370 locus in HAPE-p, HAPE-f, and HLs, its association with plasma levels of ET-1 and structural and functional stability unravelled its role in acclimatization and adaptation to HA hypoxic environment. Our investigation suggests that the rs5370 *G/T* polymorphism may be a crucial pathogenetic factor in HAPE susceptibility and a predisposing factor. Given the importance of ET1 in adaptation, endurance and maladaptation at high altitudes, the findings of this study may find therapeutic applications.

## 6. Limitation of the Study

The results of this study must be viewed in light of limitations. First, it should be noted that the study needs to be reproduced with a larger sample size to confirm the findings, although the current sample size is much higher and more competitive. Second, the inclusion of additional groups from low- and high-altitudes, perhaps ethnic, would provide further insight into the mechanisms of the rs5370 polymorphism in HA adaptation and maladaptation. Third, because HAPE is an endothelial disorder, it would be more useful to perform the ELISA assay on an endothelial cell line, such as HUVEC, even animal-based studies would be beneficial. Fourth, the *in silico* evaluation in this study provided encouraging results, yet further detailed analyses are needed to confirm the findings concerning the structure-function relationship of the protein. Fifth, considering the significant role of ET1 in athletes, this study needs to be evaluated in high-performance sports and medicine. Undoubtedly, a human physiological system is complex and multifactorial; therefore, any effect of a single genetic locus or several of its loci and the respective protein may be like a tip of an iceberg. Although, the rs5370 polymorphism is associated with raised ET1 levels in human blood tissue and cells, investigating other *EDN1* polymorphisms would be desirable and may add to the knowledge.

## Figures and Tables

**Table 1:** Genotype and allele distribution of the polymorphism rs5370 of *EDN1* in HAPE-f, HAPE-p, and HLs.

Genetic Model	rs ID	HAPE-f	HAPE-p	HLs	HAPE-f vs. HAPE-p		HAPE-f vs. HLs		HLs vs. HAPE-p	
	rs5370	Total Count (%)			OR (95% CI)	*p* Value	OR (95% CI)	*p* Value	OR (95% CI)	*p* Value
Co-dominant	GG	128 (41.2)	87 (28.0)	168 (54.1)	Ref	-	Ref	-	Ref	-
	GT	151 (48.7)	204 (65.8)	130 (41.9)	2.17 (1.52–3.10)	1.75 × 10^−5^	0.60 (0.41–0.90)	0.01	3.37 (2.27–5.01)	1.78 × 10^−9^
	TT	31 (10)	19 (6.12)	12 (3.8)	0.90 (0.47–1.72)	0.76	2.66 (1.08–6.54)	0.03	0.13 (0.14–0.71)	0.006
Dominant	GG	128 (41.2)	87 (28.0)	168 (54.1)	Ref	-	Ref	-	Ref	-
	GT+TT	182 (58.7)	223 (71.9)	142 (45.8)	1.95 (1.38–2.76)	1.39 × 10^−4^	0.56 (0.38–0.82)	0.003	3.31 (2.24–4.89)	1.57 × 10^−9^
Recessive	TT	31 (10)	19 (6.12)	12 (3.8)	Ref	-	Ref	-	Ref	-
	GG+GT	279 (90.0)	291 (93.8)	298 (96.1)	0.55 (0.30–1.01)	0.05	0.45 (0.20–0.98)	0.04	1.20 (0.52–2.79)	0.65
Allelic	G	407 (65.6)	378 (60.9)	466 (75.1)	Ref	-	Ref	-	Ref	-
	T	213 (34.4)	242 (39.1)	154 (24.9)	1.25 (0.99–1.59)	0.05	0.63 (0.47–0.84)	0.002	1.96 (1.48–2.61)	3.09 × 10^−6^

*p* values were obtained after adjustment with age and BMI by multinomial logistic regression analysis using SPSS 15.0 software. The genotype distribution and allele frequency were compared by χ^2^ tests. %, per cent distribution; Ref, reference; OR, odds ratio; CI, confidence interval; P, significance.

## Supplementary Materials

The following supporting information can be downloaded at: https://www.mdpi.com/article/10.3390/ijerph191811174/s1, Figure S1: ConSurf analysis plot; Figure S2: Frustration and configurational loss and gain contacts in K198N region. Table S1: PCR conditions of rs5370 polymorphism of *EDN1*; Table S2: qRT-PCR conditions of *EDN1*; Table S3: Clinical characteristics of the HAPE-f, HAPE-p and HLs. Table S4: System characterization for parameters after 100-ns simulation.
