# Supplementary material for: The EDN1 Missense Variant rs5370G > T Regulates Adaptation and Maladaptation under Hypobaric Hypoxia
Source: Int J Environ Res Public Health. 2022 Sep 6;19(18):11174. doi: 10.3390/ijerph191811174 (PMC9517604; doi:10.3390/ijerph191811174)
Supplement: Supplementary file 1 [file ijerph-19-11174-s001.zip › ijerph-1858991-supplementary.pdf]

Supplementary Tables

Table S1. PCR conditions of rs5370 polymorphism of *EDN1*

Table S2. qRT-PCR conditions of *EDN1*

Table S3. Clinical characteristics of the HAPE-f, HAPE-p and, HLs

Table S4. System characterization for parameters after 100-ns simulation

Supplementary figures

Figure S1. ConSurf analysis plot. The plot revealed residual conservation of the *EDN1* sequence. The rs5370 residue was located in the variable region.

Figure S2. Frustration and configurational loss and gain contacts in K198N region. (A) Panel shows density and covariances matrices of *EDN1* K198. (b) Panel depicts *EDN1* 198N density and covariances matrices type. Green shades represent minimally frustrated and red shades represent highly frustrated residues. Å, Angstrom; i and j, covariance matrix for residues; %, per cent.

Table S1. PCR conditions of rs5370 polymorphism of *EDN1*

Gene	rs ID	Primer Sequence	Cycling Conditions	Product size, bp
EDN1	rs5370G/T	F 5′-GACCATGAGAAACAGCGTCA-3′<br>R 5′-AGAGGATGCTCCTGCTCTGA-3′	I95°C 5', D95°C 30'', A60°C 40'', E72°C 40'', 35cy, FE72°C 10'	201
		IP 5'-CTAGGGTTCGACTTTCCGTT-3'	I96°C 3', D96°C 10'', A50°C 5'', E60°C 30'', 32cy, 4°C 10'	

A, annealing; cy, cycles; D, denaturation; E, extension; F, forward; FE, final extension; I, initial denaturation; IP, internal primer; R, reverse; bp, base pair.

Table S2. qRT-PCR conditions of *EDN1*

Gene	Primer sequences	Cycling conditions	Product size (bp)
EDN1	F 5' - AGCTGTCCAAGTCAGACGC - 3'<br>R 5' - GCCCAAGTGCCCTTTTAACG - 3'	I 94°C 10', D94°C 15'', A60°C 60'', 40 cy	84

A, annealing; cy, cycles; D, denaturation; E, extension; F, forward; FE, final extension; I, initial denaturation; IP, internal primer;
R, reverse; bp, base pair.

Table S3. Clinical characteristics of the HAPE-f, HAPE-p and, HLs

	HAPE-f	HAPE-p	HLs	HAPE-f vs. HAPE-p	HAPE-f vs. HLs	HAPE-p vs. HL
				***P* values**		
**n**	310	310	310			
**Age**	29.5±6.2	29.2±8.5	37.5±11.2	0.65	7.37E-22	1.49E-19
**BMI, Kg/m²**	22.1± 3.2	22.9± 13.1	21.20± 3.5	0.30	3.85E-04	0.02
**SaO_2, %**	87.7± 4.4	48.6± 13.5	87.7± 9.9	4.71E-77	8.77E-24	3.92E-65
**PR, rate/min**	84.2± 20.4	102.8± 21.9	75.4± 10.6	6.38E-09	7.44E-05	5.76E-18
**MAP, mmHg**	92.8± 7.8	95.6± 10.7	93.2± 6.91	0.02	0.85	0.02

Data are presented as mean ± SD and are compared by unpaired Student's *t*-test. n, number of subjects; BMI, body mass index; SaO_2, arterial oxygen saturation; %, per cent; PR, pulse rate; MAP, mean arterial pressure.

Table S4. System characterization for parameters after 100-ns simulation.

Type	Average RMSD (Å)	Average RMSF (Å)	Radius of gyration (nm)	Average SASA (nm²)	Average no. of Hydrogen bonds
EDN1 wild-type	2.81	0.93	2.97	130.63	110
EDN1 variant-type	2.05	0.91	3.20	129.28	114

Variant, K198N; Å, Angstrom; nm, nanometer; nm², nanometre square; SASA, solvent-accessible surface area.

Supplementary Figure S1:

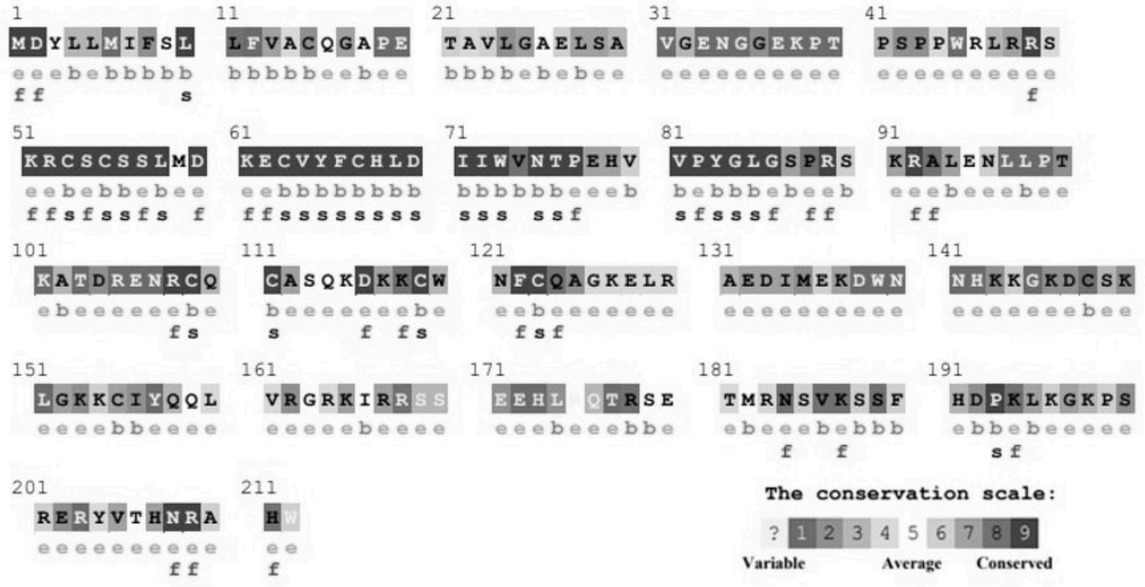

Figure S1. ConSurf analysis plot. The plot revealed residual conservation of the *EDN1* sequence. The rs5370 residue was located in the variable region.

Supplementary Figure S2:

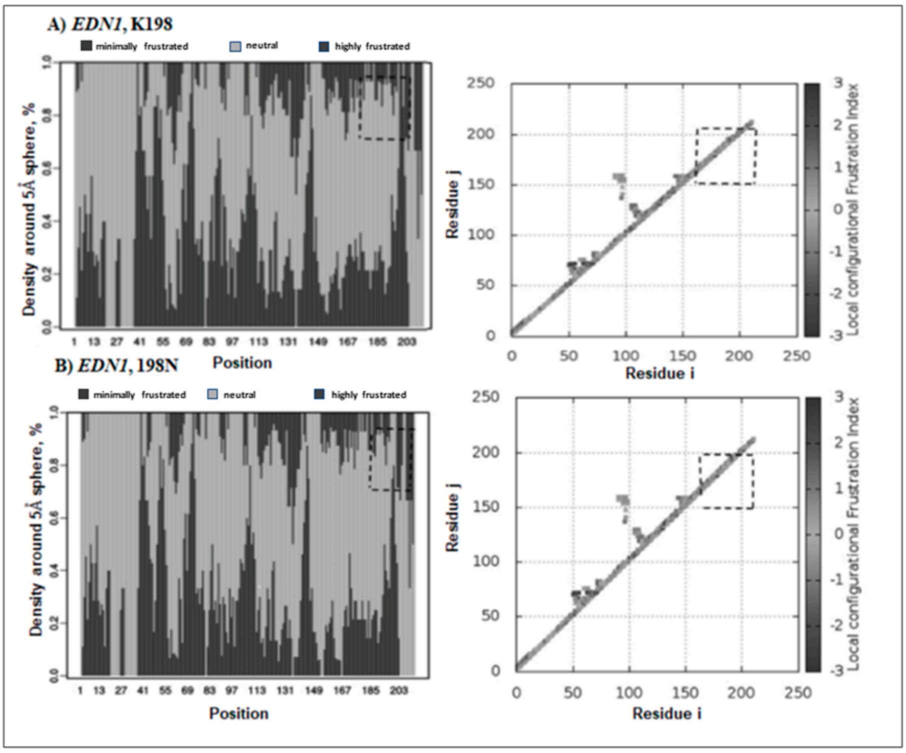

Figure S2. Frustration and configurational loss and gain contacts in K198N region. (**A**) Panel shows density and covariances matrices of *EDN1* K198. (**B**) Panel depicts *EDN1* 198N density and covariances matrices type. Green shades represent minimally frustrated and red shades represent highly frustrated residues. Å, Angstrom; i and j, covariance matrix for residues; %, per cent.
